# Poor prognosis of intra‐tumoural TRBV6‐6 variants in *EGFR*‐mutant NSCLC: Results from the ADJUVANT‐CTONG1104 trial

**DOI:** 10.1002/ctm2.775

**Published:** 2022-04-22

**Authors:** Cunte Chen, Siyang Maggie Liu, Yedan Chen, Ming Han, Qiuxiang Ou, Hua Bao, Ling Xu, Yikai Zhang, Jia‐Tao Zhang, Wenzhao Zhong, Qing Zhou, Xue‐Ning Yang, Yang Shao, Yi‐Long Wu, Si‐Yang Liu, Yangqiu Li

**Affiliations:** ^1^ Key Laboratory for Regenerative Medicine of Ministry of Education Institute of Hematology School of Medicine Jinan University Guangzhou China; ^2^ Department of Hematology First Affiliated Hospital The Clinical Medicine Postdoctoral Research Station Jinan University Guangzhou China; ^3^ Chinese Thoracic Oncology Group (CTONG) Guangzhou China; ^4^ Geneseeq Research Institute Nanjing Geneseeq Technology Inc. Nanjing China; ^5^ Guangdong Provincial Key Laboratory of Translational Medicine in Lung Cancer Guangdong Provincial People's Hospital Guangdong Academy of Medical Sciences Guangdong Lung Cancer Institute Guangzhou China; ^6^ School of Public Health Nanjing Medical University Nanjing China

Dear Editor,

Tumour‐infiltrating lymphocytes (TILs) contain T‐cell subsets, which are related to immune escape and poor clinical outcomes of cancer patients. Little is known which T‐cell receptor (TCR) clones belong to such T cells. Here, we identified that Vβ6‐6Jβ1‐3 and Vβ6‐6Jβ1‐6 are associated with poor prognosis for epidermal growth factor receptor (*EGFR*)‐mutant stage II/III non‐small‐cell lung cancer (NSCLC) patients treated with adjuvant gefitinib or chemotherapy VP (vinorelbine/cisplatin) in the ADJUVANT‐CTONG1104 trial.

EGFR tyrosine kinase inhibitor is the standard targeted therapy for *EGFR*‐mutant NSCLC patients.^1^, ^2^ For resectable *EGFR*‐mutant NSCLC patients, the ADJUVANT‐CTONG1104 trial showed that the first generation of EGFR‐TKI gefitinib could significantly improve disease‐free survival (DFS) of patients with N1/N2 lymph node metastasis.^3^ However, there is still heterogeneity in the clinical response to EGFR‐TKIs, which may be related to EGFR co‐mutations or immune checkpoint expression.^4^, ^5^ Regulatory T cells are correlated with cyclooxygenase‐2 expression and are closely associated with adverse clinical outcome of resected NSCLC.^6^ A recent study reported that TCR clone Vβ6‐6 was significantly increased in exhausted T cells at baseline of NSCLC patients treated with immune checkpoint blockade.^7^ While factors such as quantity of T cells in TILs have been shown to be prognostic, it remains of great interest to investigate whether any specific TCR clones may be prognostic or predictive of treatment efficacy.

In this study, we further characterized the TCR repertoire of patients from the ADJUVANT‐CTONG1104 trial and investigated the predictive potential of specific TCR‐β clones for prognosis as well as benefit from adjuvant gefitinib or chemotherapy in *EGFR*‐mutant NSCLC patients (Figure S1). NSCLC samples from 57 gefitinib‐treated patients and 44 chemotherapy‐treated patients were collected for TCR β gene sequencing to obtain TCR repertoires (Figure S2 and Supporting Information Materials and Methods). A total of 356 distinct TCR rearrangements were identified.^8^ Notably, Vβ6‐6Jβ1‐3 and Vβ6‐6Jβ1‐6 demonstrated statistical significance in predicting poor overall survival (OS) (FDR adjusted *p *< .05, Figure 1A). Importantly, the combination of Vβ6‐6Jβ1‐3 and Vβ6‐6Jβ1‐6 was the best model in predicting OS (Figure 1B), which was internally validated by 100 repeated 10‐fold cross‐validation (Figure S3). Multivariate Cox regression analysis indicated that Vβ6‐6Jβ1‐3 contributed the greatest to OS prediction (*p* < .001) (Figure 1C). Overall, the results indicated both Vβ6‐6Jβ1‐3 and Vβ6‐6Jβ1‐6 associated with poor OS of *EGFR*‐mutant NSCLC patients.

**FIGURE 1:**
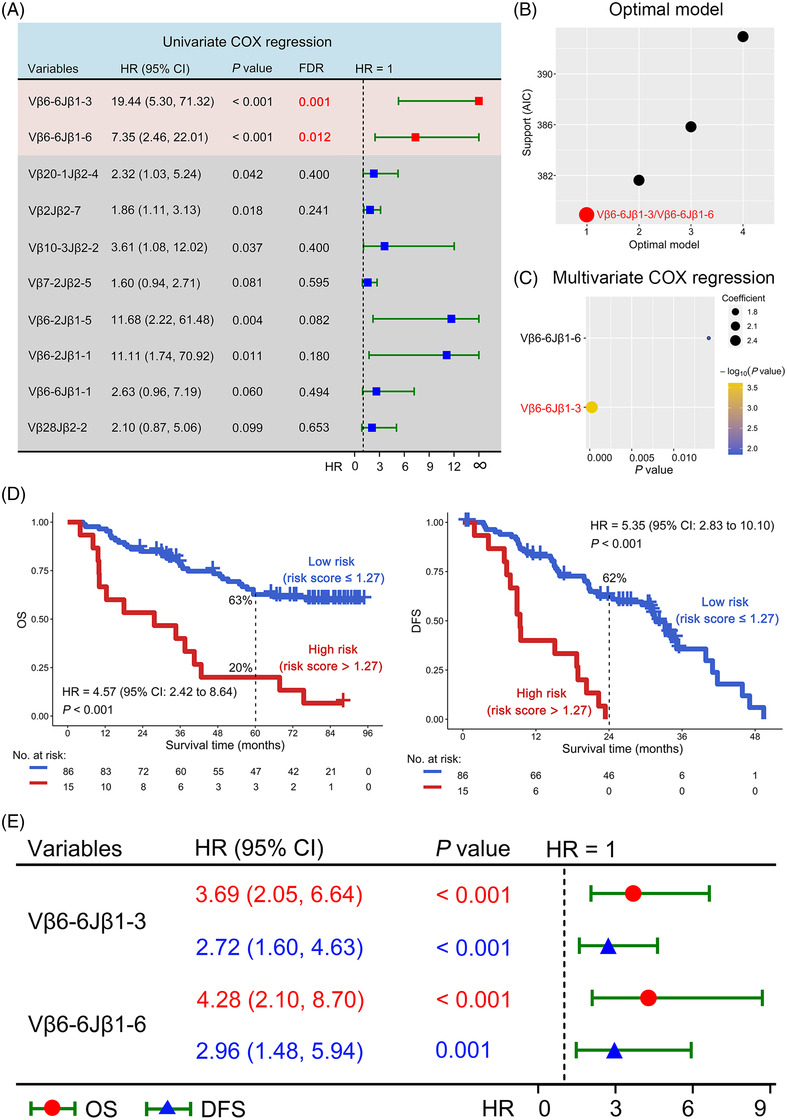
Evaluating optimal model using T‐cell receptor (TCR) rearrangements for prognostication. (A) Among of 144 high‐frequency (>0.1) TCR rearrangements, 10 TCR rearrangements were associated with poor overall survival (OS) according to *p*‐value < .1 by univariate Cox regression analysis. These 10 TCR rearrangements were the just lowest unadjusted *p*‐value among many high‐frequency TCR clones. The statistically significant TCR rearrangements were marked red with FDR adjusted *p* < .05. (B) Akaike information criterion (AIC) profile from the best to the worst model was obtained. (C) The bubble plot shows the contribution of Vβ6‐6Jβ1‐3 and Vβ6‐6Jβ1‐6 to OS. The contribution was exhibited by the coefficients β of Vβ6‐6Jβ1‐3 and Vβ6‐6Jβ1‐6 in the multivariate Cox model. The bubbles from small to large represent contributions from low to high. Based on the coefficients, the risk score was calculated as follows: Risk score = 2.68* (frequency of Vβ6‐6Jβ1‐3) + 1.53* (frequency of Vβ6‐6Jβ1‐6). (D) Association between Vβ6‐6Jβ1‐3 and Vβ6‐6Jβ1‐6 combination based risk score and OS or disease‐free survival (DFS) in EGFR‐mutant non‐small‐cell lung cancer (NSCLC) patients. (E) Prognostic analysis of Vβ6‐6Jβ1‐3 and Vβ6‐6Jβ1‐6 was performed on all individuals. The red mark indicates OS analysis, whereas the blue mark indicates DFS analysis. DFS was defined as the span from randomization to recurrence or death. HR was defined as the hazard in the high‐risk group divided by the hazard in the low‐risk group. Abbreviations: CI, confidence interval; FDR, false discovery rate

Next, the ability of the optimal Vβ6‐6Jβ1‐3 and Vβ6‐6Jβ1‐6 TCR rearrangement combination model to predict OS or DFS was evaluated. Of note, the risk score was negatively correlated with OS and DFS (*p* < .001; Figure 1D, Figure S4 and Table S1). Similarly, patients with high frequency either Vβ6‐6Jβ1‐3 or Vβ6‐6Jβ1‐6 had significantly poor OS and DFS (*p *< .01, Figure 1E and Figure S5). Interestingly, hazard ratios from Vβ6‐6Jβ1‐3 and Vβ6‐6Jβ1‐6 individually were smaller compared to hazard ratios derived from the optimal combination model for both OS and DFS. These results not only show that specific TCR rearrangements (Vβ6‐6Jβ1‐3 and Vβ6‐6Jβ1‐6) are prognostic on their own, but the optimal model with a combination of these TCRs have the greatest predictive potential for prognosis of *EGFR*‐mutant NSCLC patients.

To confirm the clonotypes of TCRs, we explored the nucleotide (NT) and amino acid (AA) sequences of Vβ6‐6Jβ1‐3 and Vβ6‐6Jβ1‐6 in the high‐frequency TCR groups. The NTs and AAs at both ends other than middle in the CDR3 region were almost conserved (Figure 2A,B). Herein, we identified the top five CDR3 motifs of Vβ6‐6Jβ1‐3 and Vβ6‐6Jβ1‐6 in the high‐frequency group, which might reflect the common CDR3 sequences that contribute to the immune escape of *EGFR*‐mutated NSCLC in this study. The top five CDR3 motifs for Vβ6‐6Jβ1‐3 were YSGS, YSRS, YSIS, TLPA, and YAGS. For Vβ6‐6Jβ1‐6, the top five CDR3 motifs were YSESD, DRDGG, YSGGG, YSREG, and PRGSP (Figure 2A,B). In addition, we used TKI‐Gefitinib and Chemo‐VP arms as training and validation cohorts, and the results indicated that Vβ6‐6Jβ1‐3 and Vβ6‐6Jβ1‐6 predicted poor OS and DFS for resected *EGFR*‐mutant patients (*p* ≤ .05, Figure 3A–C). When patients carried high frequency of Vβ6‐6Jβ1‐3 or Vβ6‐6Jβ1‐6, the application of Vβ6‐6 antibody or other treatment options should be considered.

**FIGURE 2:**
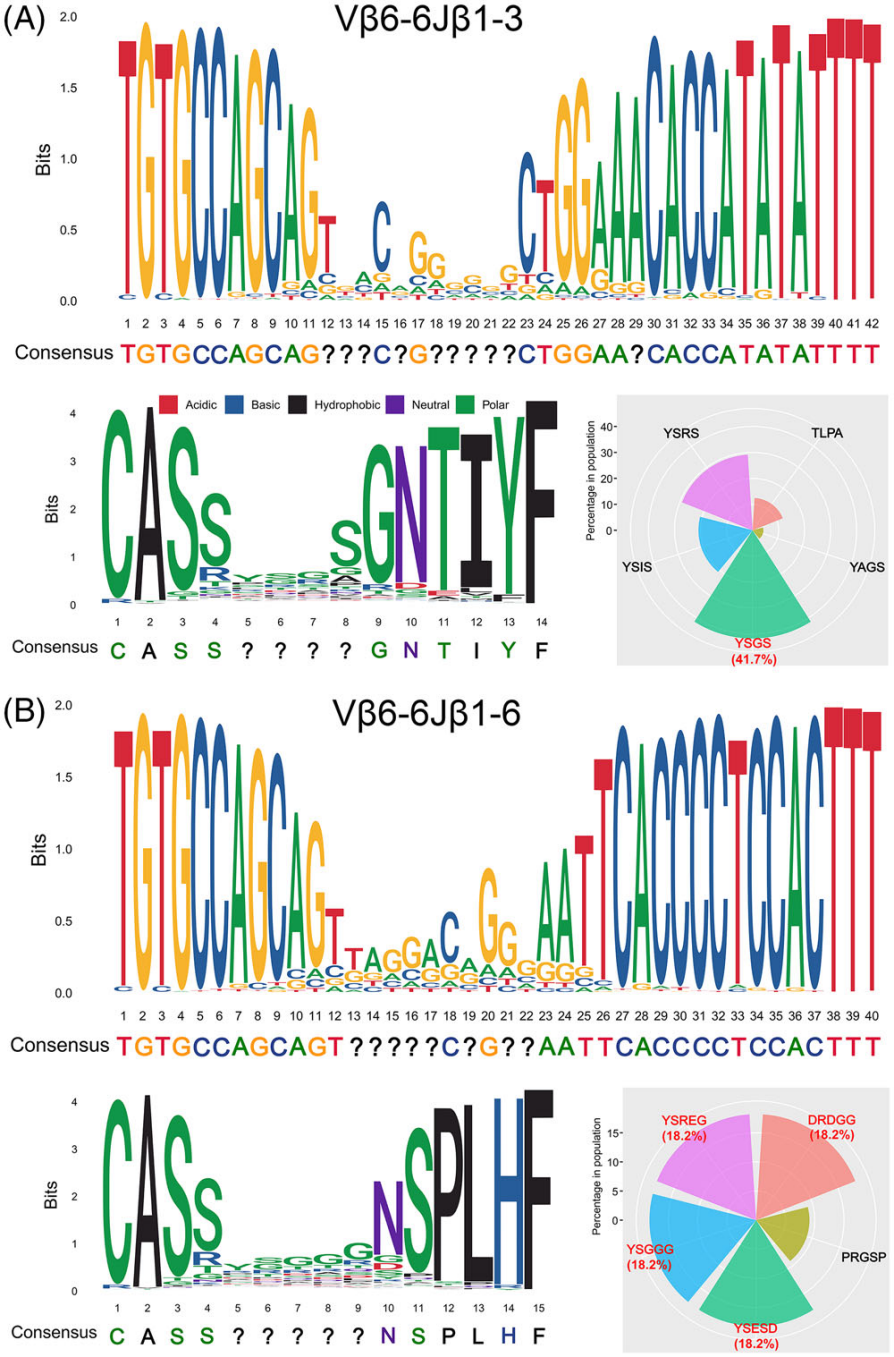
Sequence motifs of high‐frequency groups of Vβ6‐6Jβ1‐3 and Vβ6‐6Jβ1‐6 were identified. (A and B) The conservative base (upper panel) and amino acid (lower left panel) sequences of Vβ6‐6Jβ1‐3 (A) and Vβ6‐6Jβ1‐6 (B) were evaluated by local alignment using the “msa” package. The question mark “?” represents a non‐conserved base or amino acid sequence at that position. The radar plot shows the proportion of the top five non‐conserved amino acid sequences (as indicated by question marks “?”) in patients (lower right panel). The areas from small to large indicate proportions from low to high. The top five CDR3 motifs for Vβ6‐6Jβ1‐3 were YSGS (41.7%), YSRS (29.2%), YSIS (20.8%), TLPA (12.5%), and YAGS (4.2%). For Vβ6‐6Jβ1‐6, the top five CDR3 motifs were YSESD (18.2%), DRDGG (18.2%), YSGGG (18.2%), YSREG (18.2%), and PRGSP (9.1%)

**FIGURE 3:**
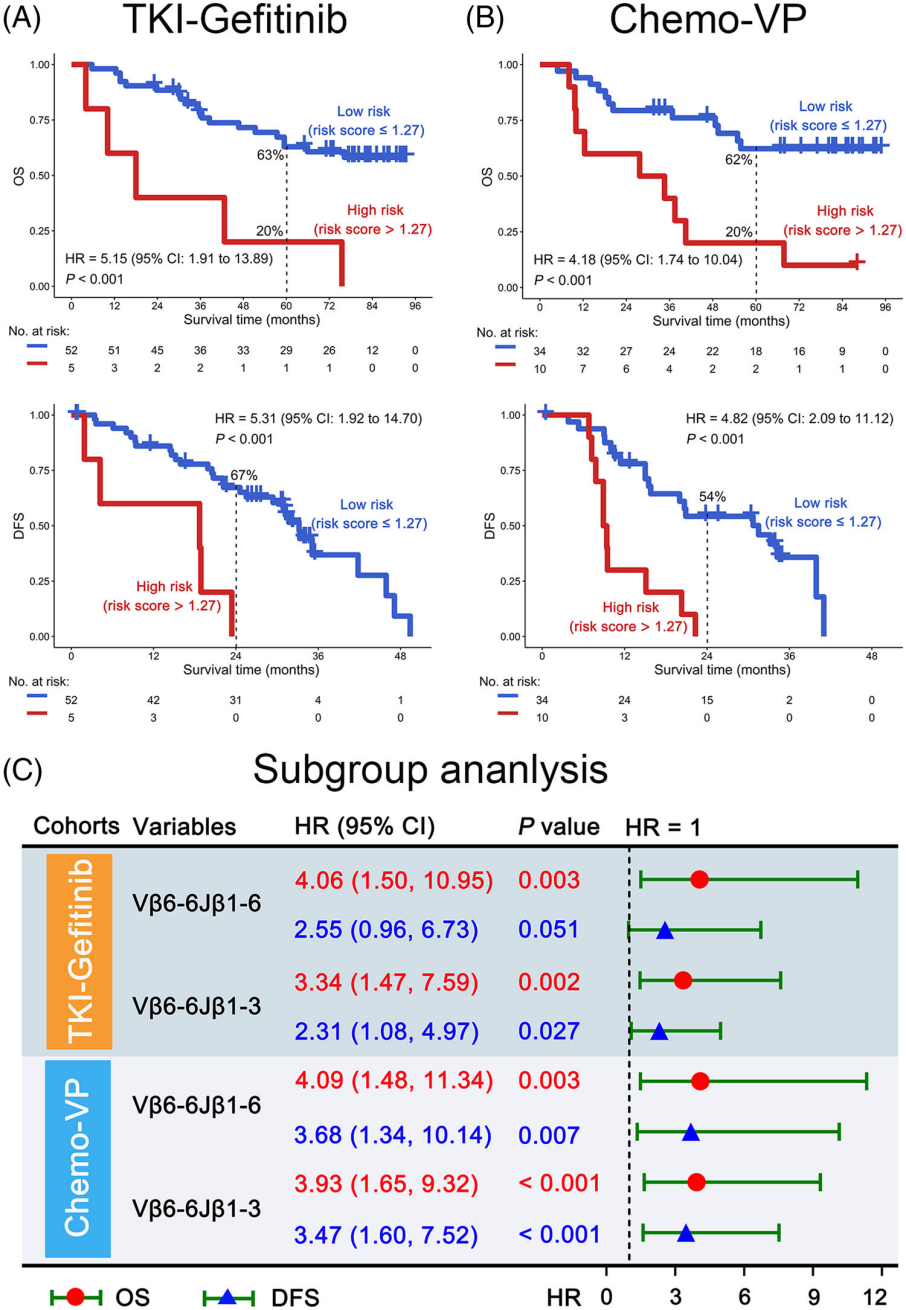
Subgroup analysis of T‐cell receptor (TCR) rearrangements. (A and B) Overall survival (OS) (upper panel) and disease‐free survival (DFS) (bottom panel) analysis of risk score estimated by the coefficients β of Vβ6‐6Jβ1‐3 and Vβ6‐6Jβ1‐6 in the multivariate Cox model in the TKI‐Gefitinib (A) and Chemo‐VP (B) cohorts. (C) Prognostic analysis of Vβ6‐6Jβ1‐3 and Vβ6‐ 6Jβ1‐6 individually in TKI‐Gefitinib and Chemo‐VP cohorts. The red mark indicates OS analysis, whereas the blue mark indicates DFS analysis

Mutant peptides produced by somatic tumour‐specific mutations may create a neoepitope on cancer cells, which can be recognized by T cells. Therefore, the correlation of TCR rearrangements with genes with alteration rates greater than 20% was explored (Figure S6). Higher frequency of Vβ6‐6Jβ1‐3 rather than Vβ6‐6Jβ1‐6 was found in patients with *NKX2‐1* copy number (CN) gain, with marginal significance (*p* = .058), and they were significantly positively correlated (Cramer's *V* = 0.27, *p* = .007) (Figure 4A and Figure S7A). Furthermore, no significant relationship was found between Vβ6‐6Jβ1‐3 or Vβ6‐6Jβ1‐6 and *TP53* exon 4/5 missense (*p *> .05, Figure S7B,C). Previously, we found *NKX2‐1* CN gain was significantly associated with poor prognosis of *EGFR*‐mutant stage II/III NSCLC patients.^9^ *NKX2‐1* also serves an essential role in determining the fate of lung cancer cells and shaping the tumour immune microenvironment.^10^ Hence, results here appear to be consistent with previous findings, and demonstrated cross‐talk between mutational and immune landscape. Compared to patients who were Vβ6‐6Jβ1‐3^low^ and *NKX2‐1* wild type, patients with either Vβ6‐6Jβ1‐3^high^ or *NKX2‐1* CN gain or both have a shorter OS or DFS, especially in Chemo‐VP cohort (*p *< .05, Figure 4B and Figure S8A–C). Taken together, clonally expanded Vβ6‐6Jβ1‐3 and *NKX2‐1* CN gain may be an important biomarker for guiding adjuvant chemotherapy decisions in resectable early‐stage NSCLC patients.

**FIGURE 4:**
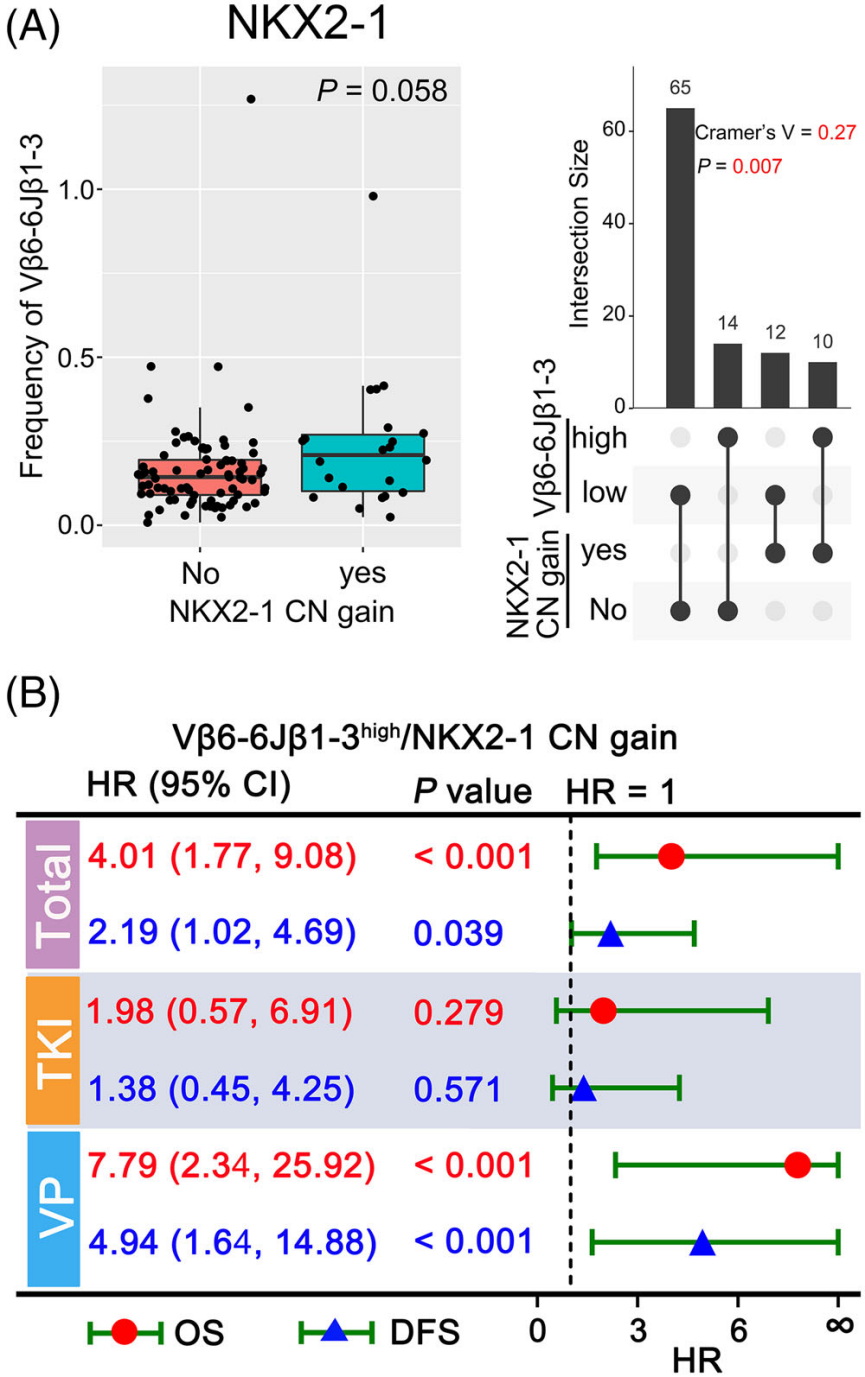
Correlation between Vβ6‐6Jβ1‐3 and *NKX2‐1* (NK2 homeobox 1) copy number (CN) gain. (A) The frequency distribution of Vβ6‐6Jβ1‐3 between patients with and without *NKX2‐1* CN gain (left panel), and the correlation between them (right panel). The optimal cut‐point for Vβ6‐6Jβ1‐3 was determined by maximally selected rank statistics, which divided patients into low‐ and high‐frequency groups. Cramer's *V* correlation was obtained by chi‐square test. The closer the value of Cramer's *V* is to –1 or 1, the stronger the correlation. (B) The association between Vβ6‐6Jβ1‐3high/*NKX2‐1* CN gain and OS or DFS. The red mark represents OS analysis, whereas the blue mark represents DFS analysis

In summary, we identified Vβ6‐6Jβ1‐3 and Vβ6‐6Jβ1‐6 in TILs, which were significantly correlated with poor prognosis in *EGFR*‐mutant NSCLC patients in an adjuvant gefitinib or chemotherapy setting using a clinical trial cohort. To our best knowledge, this is the first study to identify specific TCR clone biomarkers related to poor clinical outcomes, as opposed to favourable outcomes. Results here are valuable for future prospective clinical trials and provide information for development of immunotherapy for *EGFR*‐mutant stage II/III NSCLC patients.

Two brief points are as follows:

1. characterizing poor prognostic TCR clones from intra‐tumoural T cells in *EGFR*‐mutant stage II/III NSCLC patients;
2. concurrent high‐frequency Vβ6‐6Jβ1‐3 and *NKX2‐1* CN gain predicted poor prognosis of *EGFR*‐mutant stage II/III NSCLC patients, especially in the adjuvant chemotherapy setting.

## CONFLICT OF INTEREST

Yi‐Long Wu discloses the following personal financial interests: Consulting and advisory services, speaking engagements of Roche, AstraZeneca, Eli Lilly, Boehringer Ingelheim, Sanofi, MSD, and BMS. The other authors have no conflict of interest.

## Supporting information



Supporting Information

Supporting Information
