# Supplementary material for: Poor prognosis of intra‐tumoural TRBV6‐6 variants in EGFR‐mutant NSCLC: Results from the ADJUVANT‐CTONG1104 trial
Source: Clin Transl Med. 2022 Apr 22;12(4):e775. doi: 10.1002/ctm2.775 (PMC9029017; doi:10.1002/ctm2.775)
Supplement: Supplementary file 1 — Supporting Information [file CTM2-12-e775-s001.doc]

**Poor prognosis of intra-tumoral TRBV6-6 variants in *EGFR*-mutant non-small cell lung cancer: results from the ADJUVANT-CTONG1104 trial**

**MATERIALS AND METHODS**

**NSCLC Patients**

Formalin-fixed and paraffin-embedded (FFPE) blocks of tumor tissues were prospectively collected from 57 patients treated with gefitinib and 44 patients treated with VP (vinorelbine/cisplatin) chemotherapy in the ADJUVANT-CTONG1104 trial.^1-4^ The median follow-up time for surviving patients was 82 months (range: 23 to 95 months). The clinical information was described in previously study.^5^

**TCRβ V Region (TRBV) Gene Sequencing**

FFPE tumor tissues of *EGFR*-mutant NSCLC patients were collected for DNA extraction and multiplex polymerase chain reaction (PCR) to construct TCR libraries. TCR libraries were then sequenced using the Illumina HiSeq 4000 platform according to the manufacturer’s instructions to obtain TCR repertoires. A multiplex PCR reaction was performed to construct TCR libraries using the QIAGEN Multiplex PCR Plus Kit with customized TCR primers, including 51 forward primers complementary to the V gene segments and 13 reverse primers complementary to the J gene segment. Additionally, 663 barcoded synthetic templates were added in the PCR reactions to calibrate for amplification bias. Amplified synthetic products and tumor samples were then purified using the AxyPrep MAG FragmentSelect-I Kit (Axygen) and the KAPA Hyper Prep Kit (KAPA Biosystems) was used for library preparation. Purified TCR libraries were then sequenced using the Illumina HiSeq 4000 platform according to the manufacturer’s instructions.

**TCR Analysis and Profiling**

For TCR profiling, fastq files were first processed to remove adapters and low-quality reads with trimmomatic and Non-V-J paired reads were further removed by Cutadapt (V 1.18). Next, paired-end read merger (PEAR, V 0.9.10) was employed to merge paired reads and synthetic standards and tumor samples were further cleaned using corresponding barcodes. Clean reads were subsequently assembled using MiXCR (V 2.1.11). Reads were aligned to reference V or J gene segments according to the international ImMunoGeneTics (IMGT) database. The final TCR repertoire contained normalized sample V/J counts, CDR3 counts, and clonality counts using synthetic standards. Analysis for TCR diversity and clonality were done as previously described with the R package vegan.^6^

**Statistical Analysis**

All statistical analyses were conducted by R software (version 4.0.2, <https://www.r-project.org/>). Univariate and multivariate Cox regression analyses were performed by the package "survival".^7^ The best model was obtained by the package "glmulti".^8^ The optimal cut-points for quantitative data were determined by maximally selected rank statistics in the package "maxstat".^9^ This is an outcome-oriented method that determines a cut-point for best separating survival outcomes.^10, 11^ The log-rank test was used to compare the Kaplan-Meier curves. The consensus of nucleotide and amino acid sequences was aligned and calculated by the package "msa".^12^ Differences between the two groups of quantitative variables were compared by the Mann-Whitney-Wilcoxon test, while the comparison of qualitative variables was performed by the Chi-square test. The area under curve (AUC) in the Receiver operating characteristic curve (ROC) was obtained with the package "pROC" ^13^. A two-tailed *P* < 0.05 was considered statistically significant.

**REFERENCES**
