# Supplementary material for: Poor prognosis of intra‐tumoural TRBV6‐6 variants in EGFR‐mutant NSCLC: Results from the ADJUVANT‐CTONG1104 trial
Source: Clin Transl Med. 2022 Apr 22;12(4):e775. doi: 10.1002/ctm2.775 (PMC9029017; doi:10.1002/ctm2.775)
Supplement: Supplementary file 2 — Supporting Information [file CTM2-12-e775-s002.docx]

**Poor prognosis of intra-tumoral TRBV6-6 variants in *EGFR*-mutant non-small cell lung cancer: results from the ADJUVANT-CTONG1104 trial**


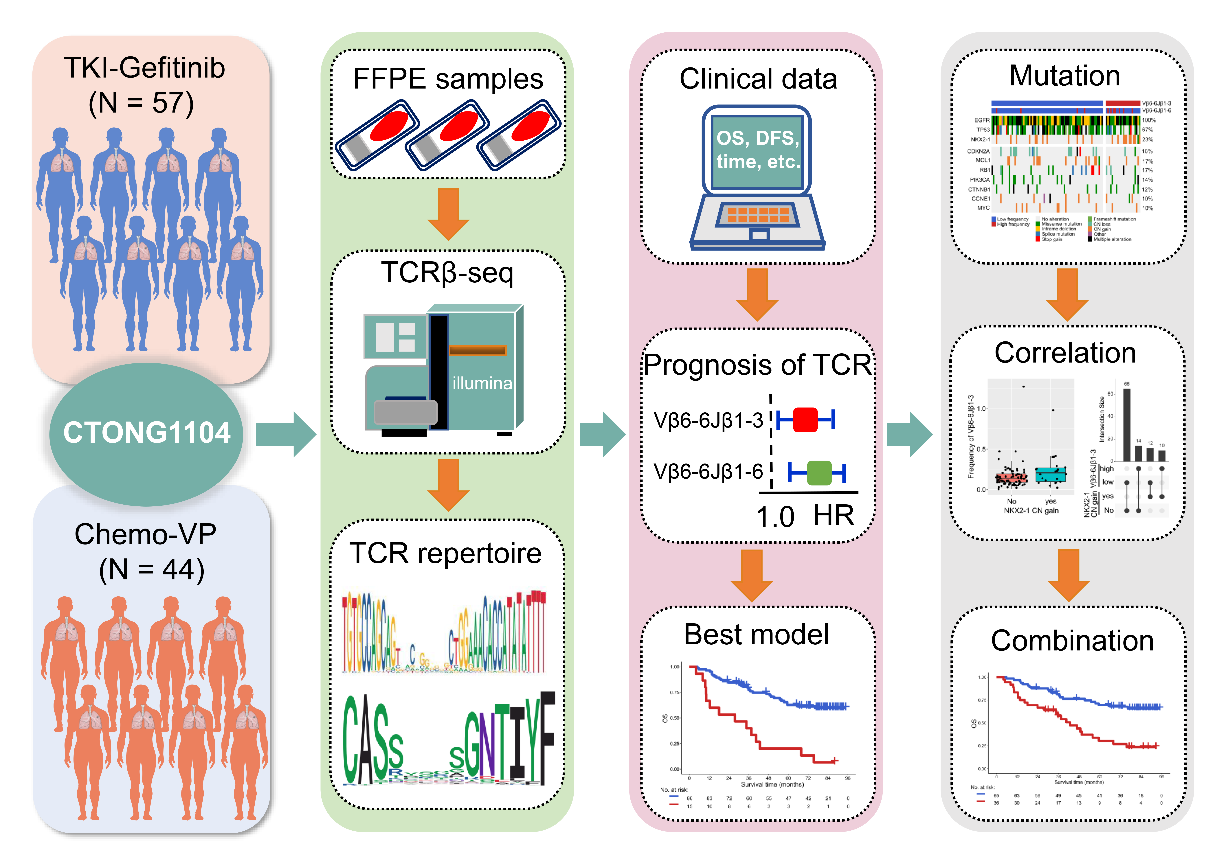


**Figure S1.** Study design. Stage II/III NSCLC patients with an epidermal growth factor receptor (*EGFR*) mutation from the ADJUVANT-CTONG 1104 trial were divided into two cohorts and received EGFR tyrosine kinase inhibitor gefitinib (TKI-Gefitinib, n = 57) or vinorelbine/cisplatin (Chemo-VP, n = 44) treatment. Formalin-fixed and paraffin-embedded (FFPE) blocks of tumor tissues were used for T cell receptor β-chain (TCRβ) sequencing to identify TCR repertoire. Associations between TCR rearrangements and clinical outcomes or mutated genes were investigated. HR: hazard ratio.


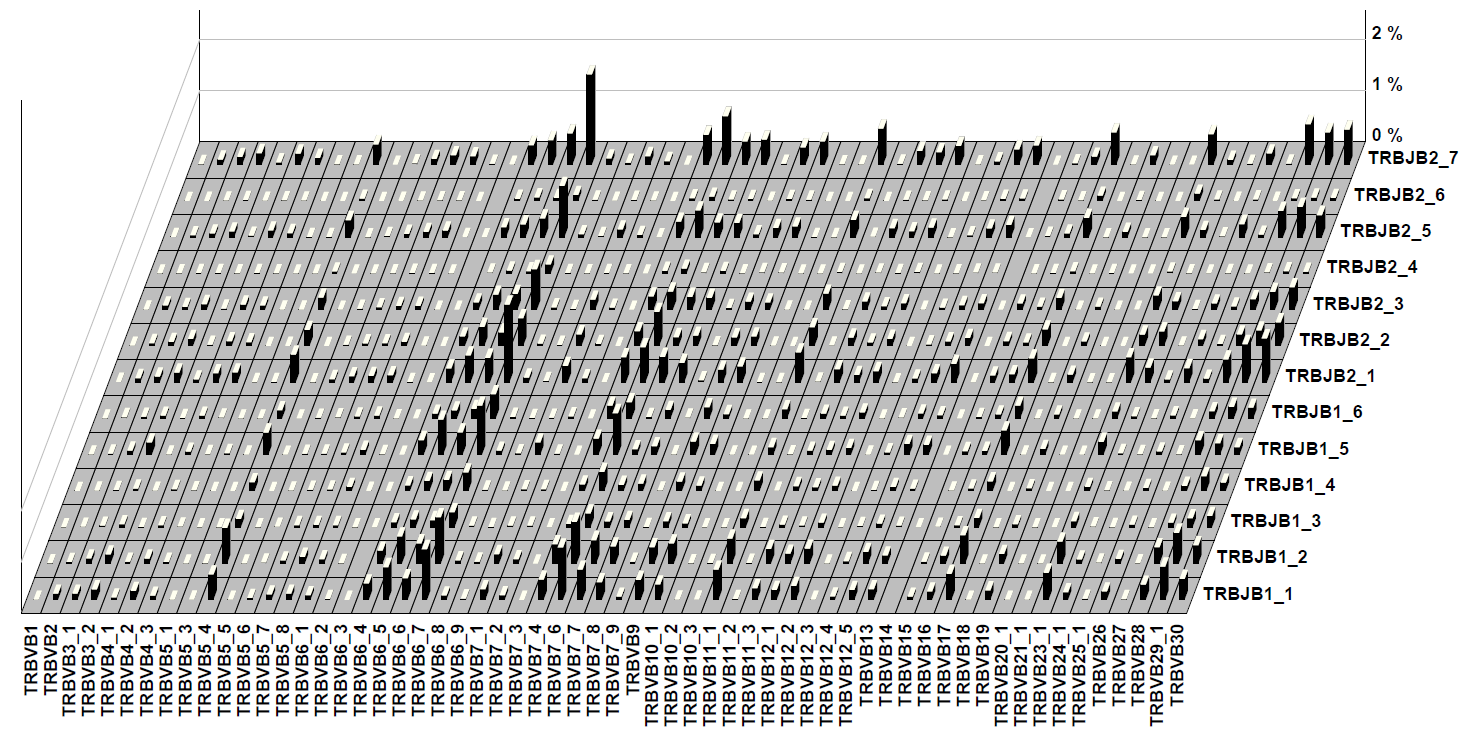


**Figure S2.** 3D bar plot illustrating the average percentage usage of all possible TCR Vbeta/Jbeta combinations in the study cohort.


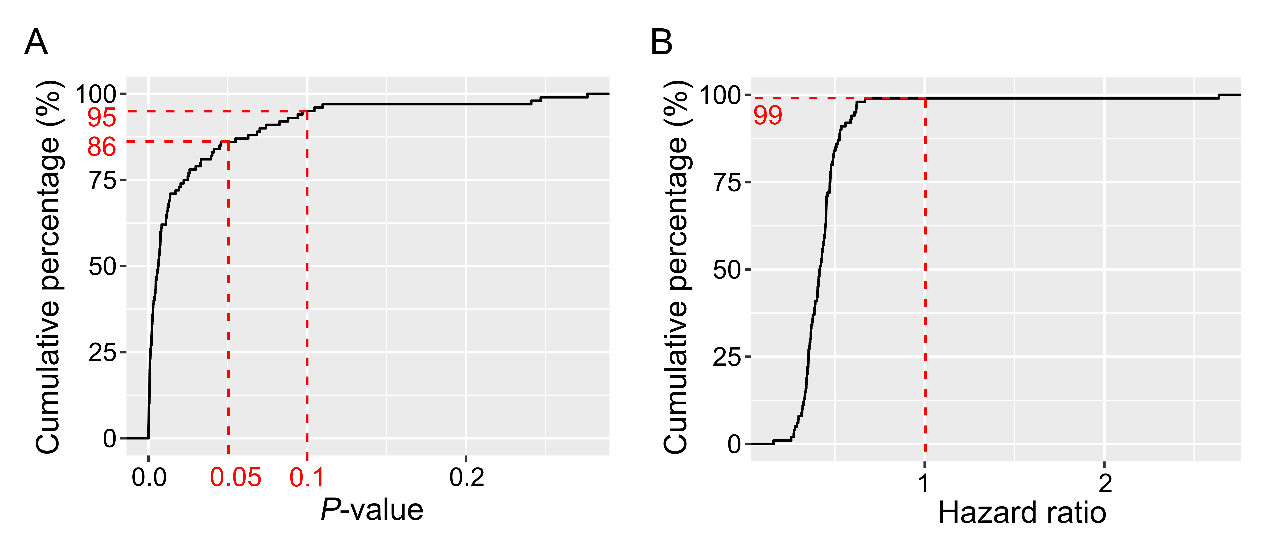


**Figure S3.** Internal validation of risk score. (A) Curve showing the cumulative percentage of *P*-values from 100 repeated 10-fold cross validation of the same VJ selection criteria and risk score algorithm. Corresponding *P*-values were derived by comparing overall survival difference between high and low risk score groups. Red dotted lines indicate percentage of repeats with *P* < 0.05 and <0.1 (two-sided, wald test). (B) Curve showing the cumulative percentage of hazard ratios from 100 repeated 10-fold cross validation, derived by comparing overall survival difference between high and low risk groups. Red dotted line indicates percentage of repeats with hazard ratio >1.


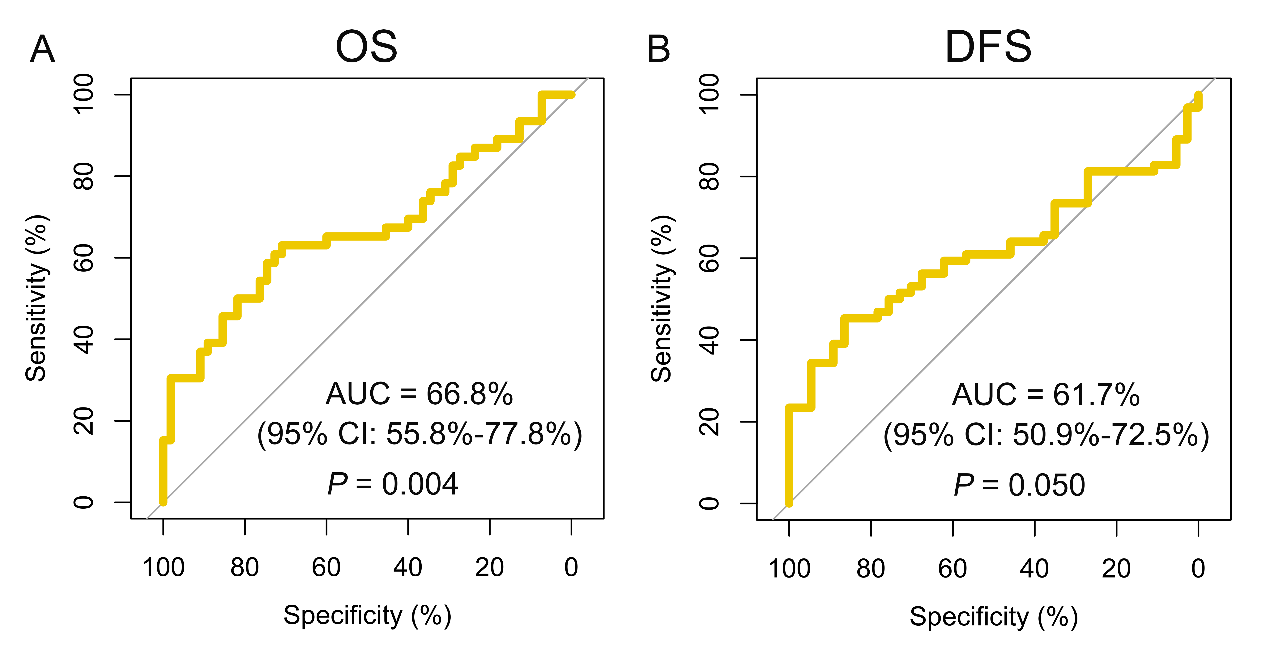


**Figure S4.** Receiver operating characteristic curves (ROCs) were used to evaluate the performance of the combined model in the prediction of OS (A) and DFS (B). The ROC curves suggested that Vβ6-6Jβ1-3 and Vβ6-6Jβ1-6 TCR rearrangement combination model had a good performance in the prediction of death (OS: AUC = 66.8%, 95% CI: 55.8%-77.8%, *P* = 0.004). Similar results were found in the prediction of disease recurrence or death (DFS: AUC = 61.7%, 95% CI: 50.9%-72.5%, *P* = 0.050). AUC: Area under curve.


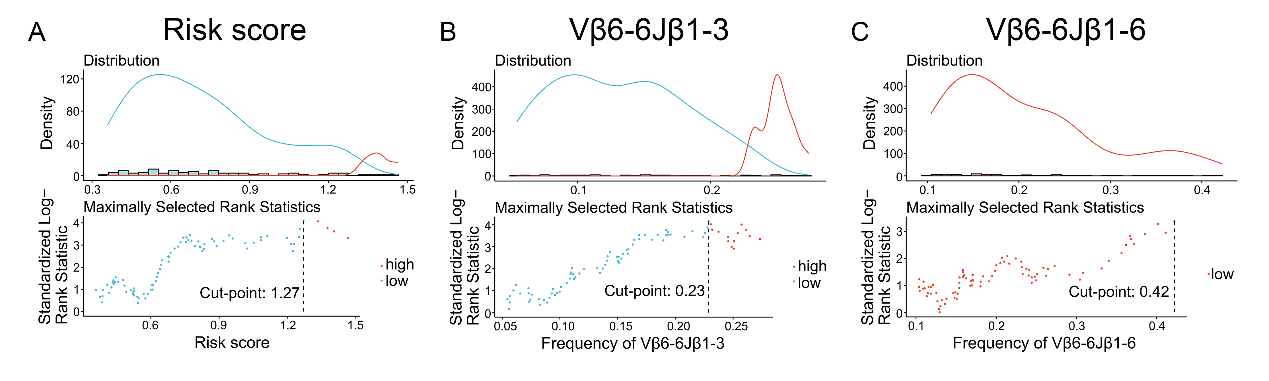


**Figure S5.** The optimal cut-points for combination risk score (A), Vβ6-6Jβ1-3 (B), and Vβ6-6Jβ1-6 (C) individually were determined by the "survminer" package in R (version 4.0.2).


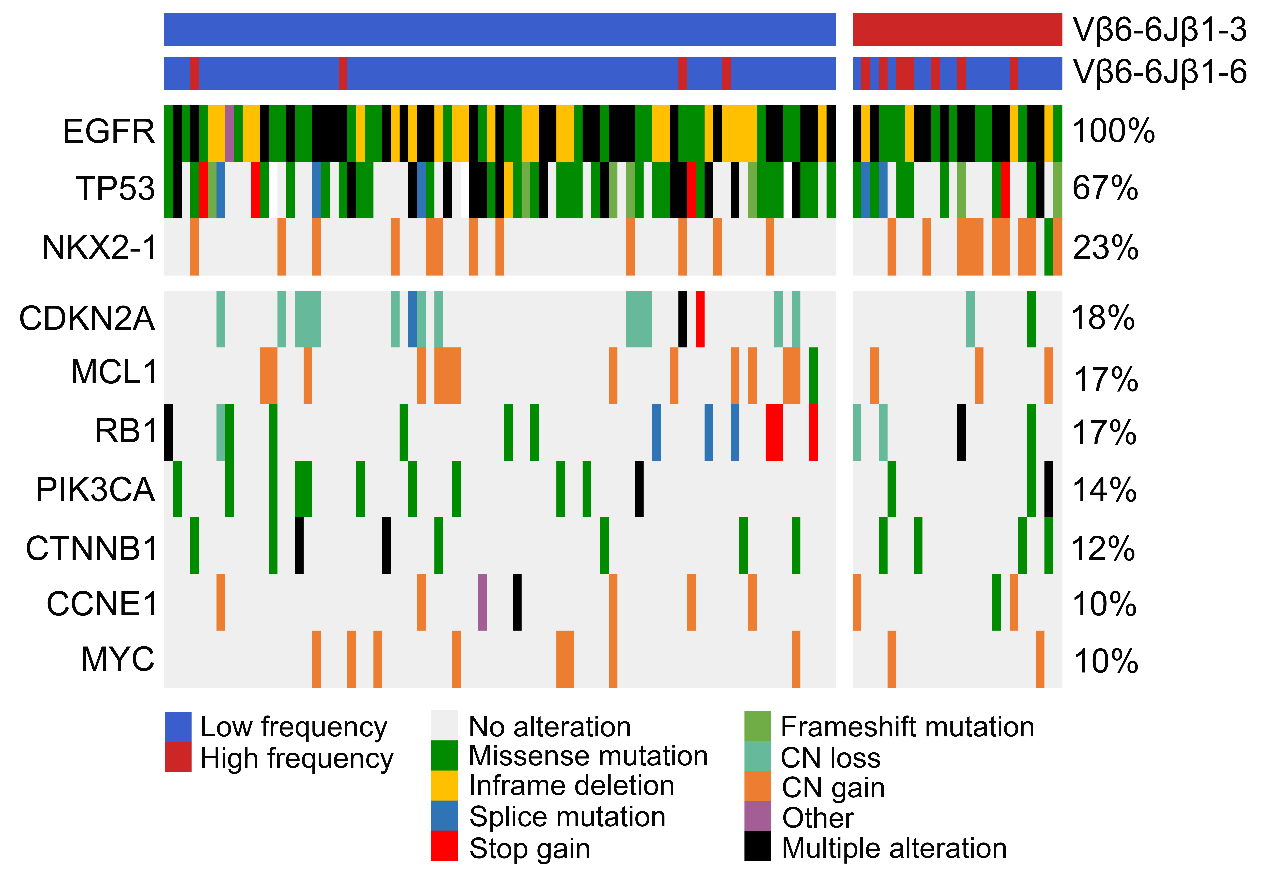


**Figure S6.** The waterfall plot shows the landscape of the top 10 mutated genes in stage II/III NSCLC patients with an *EGFR* mutation.


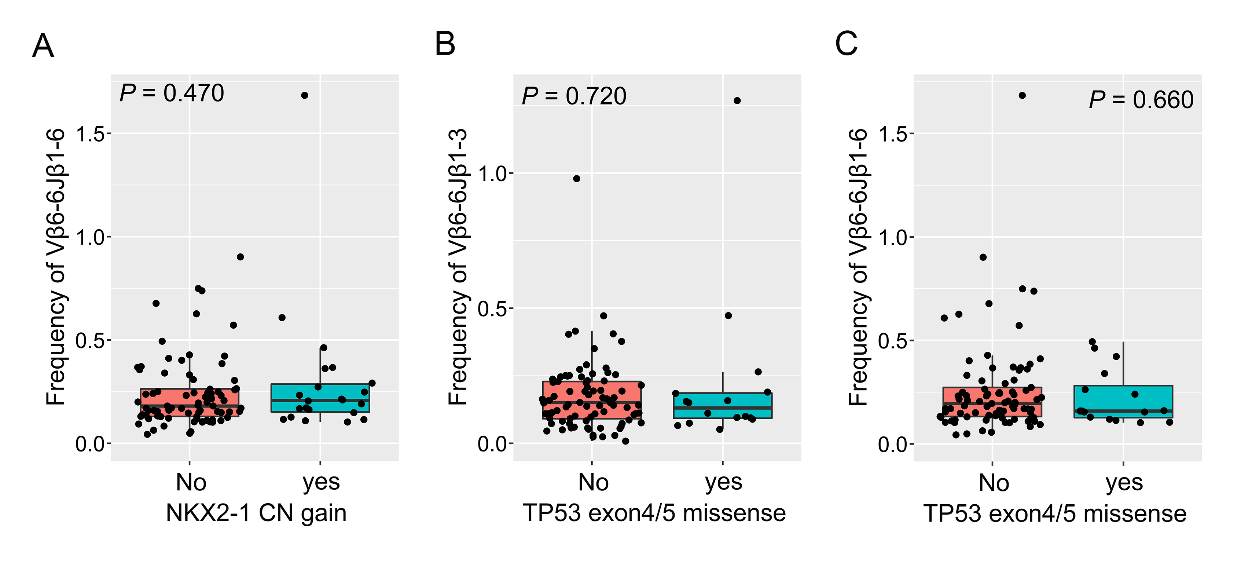


**Figure S7.** Correlation between Vβ6-6 and genetic alteration. (A and C) Correlation between Vβ6-6Jβ1-6 and *NKX2-1* CN gain (A) or *TP53* exon4/5 missense (C). (B) The frequency distribution of Vβ6-6Jβ1-3 between those with and without *TP53* exon4/5 missense.


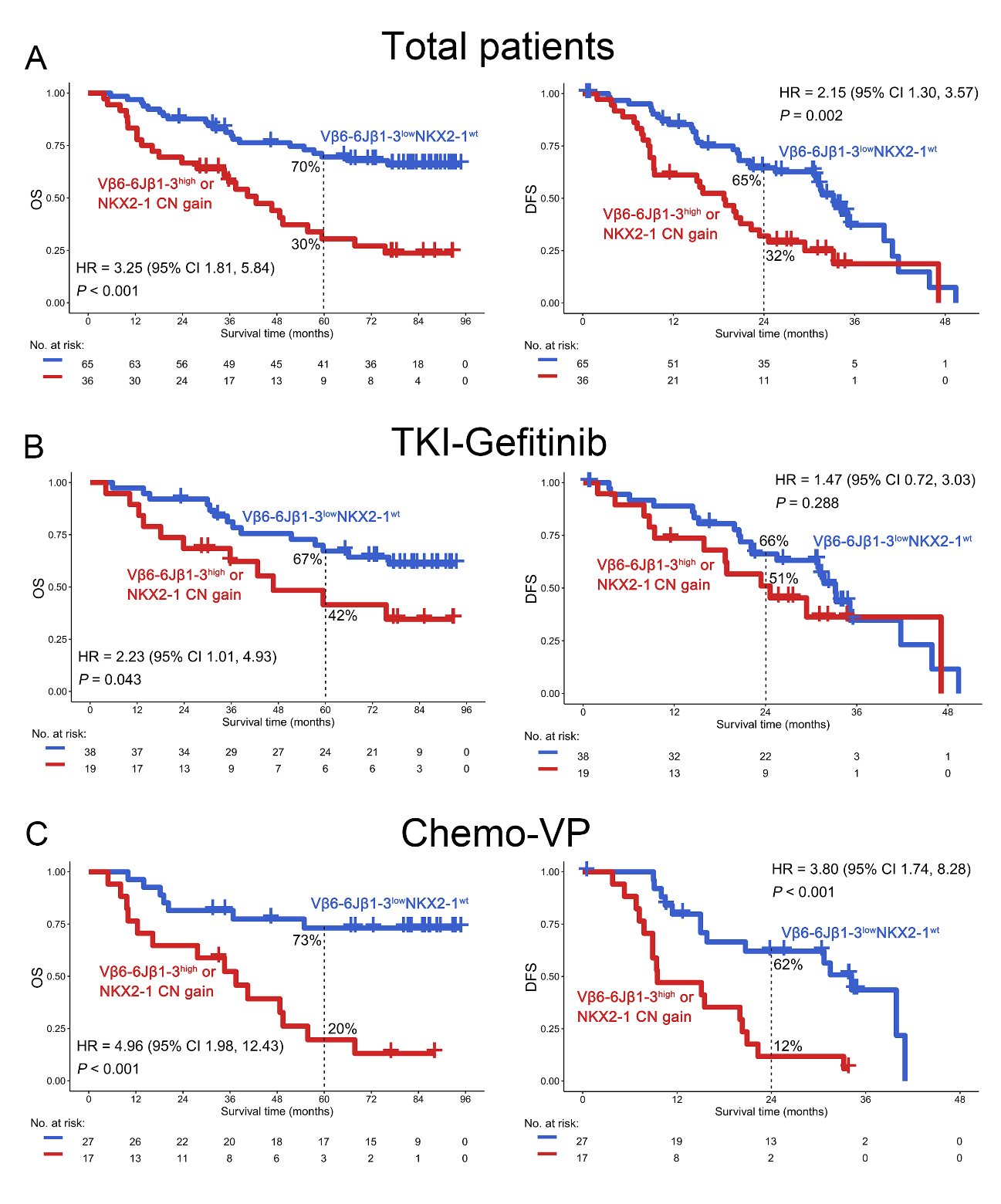


**Figure S8.** OS and DFS analysis of patients with Vβ6-6Jβ1-3^low^NKX2-1^wt^ and Vβ6-6Jβ1-3^high^ or NKX2-1 CN gain or both in all patients (A), and TKI-Gefitinib (B), Chemo-VP (C) cohorts.

**Table S1.** Univariate and multivariate COX regression analysis in *EGFR*-mutant NSCLC patients.

| Variables | OS | | | | |  | DFS | | | | |
| --- | --- | --- | --- | --- | --- | --- | --- | --- | --- | --- | --- |
|  | Univariate COX | |  | Multivariate COX | |  | Univariate COX | |  | Multivariate COX | |
|  | HR  (95% CI) | *P* value |  | HR  (95% CI) | *P* value |  | HR  (95% CI) | *P* value |  | HR  (95% CI) | *P* value |
| Risk score  (ref: Low risk) | |  |  |  |  |  |  |  |  |  |  |
| High risk | 4.57  (2.42, 8.63) | < 0.001 |  | 5.61  (2.65, 11.84) | < 0.001 |  | 5.33  (2.82, 10.07) | < 0.001 |  | 5.03  (2.51, 10.10) | < 0.001 |
| Sex (ref: Female) | |  |  |  |  |  |  |  |  |  |  |
| Male | 1.03  (0.58, 1.85) | 0.918 |  | 1.38  (0.60, 3.21) | 0.450 |  | 1.24  (0.74, 2.08) | 0.416 |  | 1.99  (0.88, 4.53) | 0.100 |
| Age (years) | 1.02  (0.98, 1.05) | 0.316 |  | 1.03  (0.99, 1.06) | 0.109 |  | 1.01  (0.98, 1.04) | 0.713 |  | 1.00  (0.97, 1.03) | 0.878 |
| Smoking history  (ref: Never) | |  |  |  |  |  |  |  |  |  |  |
| Ever | 1.31  (0.54, 3.13) | 0.552 |  | 1.18  (0.39, 3.61) | 0.772 |  | 1.33  (0.62, 2.83) | 0.465 |  | 2.91  (1.02, 8.32) | 0.046 |
| Current | 1.77  (0.84, 3.73) | 0.132 |  | 2.47  (0.91, 6.68) | 0.076 |  | 1.19  (0.60, 2.37) | 0.624 |  | 2.39  (0.89, 6.44) | 0.085 |
| Pathology  (ref: Adenocarcinoma) | |  |  |  |  |  |  |  |  |  |  |
| Other | 0.99  (0.24, 4.07) | 0.985 |  | 1.24  (0.28, 5.56) | 0.776 |  | 1.31  (0.47, 3.68) | 0.608 |  | 1.85  (0.60, 5.69) | 0.284 |
| Clinical stage (ref: II) | |  |  |  |  |  |  |  |  |  |  |
| III | 1.38  (0.72, 2.62) | 0.332 |  | 0.00  (0.00, >50) | 0.927 |  | 1.51  (0.86, 2.63) | 0.149 |  | 2.01  (0.26, 15.92) | 0.507 |
| N stage (ref: N1) | |  |  |  |  |  |  |  |  |  |  |
| N2 | 1.47  (0.77, 2.79) | 0.246 |  | >50  (0.00, >50) | 0.926 |  | 1.49  (0.86, 2.58) | 0.156 |  | 0.73  (0.09, 5.58) | 0.758 |
| Treatment (ref: VP) | |  |  |  |  |  |  |  |  |  |  |
| TKI | 0.84  (0.47, 1.50) | 0.560 |  | 1.17  (0.62, 2.19) | 0.633 |  | 0.62  (0.37, 1.02) | 0.061 |  | 0.59  (0.33, 1.05) | 0.073 |

CI: confidence interval; DFS: disease-free survival; HR: hazard ratio; OS: overall survival; TKI: tyrosine kinase inhibitor; VP: vinorelbine plus cisplatin.
